# Multiscale Petrophysical Response of Sandstone and Limestone Formations During CO_2_ Sequestration

**DOI:** 10.1021/acsomega.6c03419

**Published:** 2026-08-03

**Authors:** Valentina Parra-Henao, Julian Anaya-Florez, Nicolás Santos-Santos, Jimena Gómez-Delgado

**Affiliations:** 28014 Universidad Industrial de Santander, 680002 Bucaramanga, Colombia

## Abstract

The injection of CO_2_ into deep geological formations induces geochemical interactions that may modify reservoir petrophysical properties and influence the long-term performance of carbon capture and storage (CCS) projects. This experimental study evaluates the effects of CO_2_ injection on sandstone and limestone through an integrated petrophysical and physicochemical characterization, including X-ray diffraction (XRD), computed tomography (CT), coreflooding experiments, and fluid compatibility analyses. A distinguishing aspect of this research lies in its focus on the Mugrosa Formation (Colombia), using a representative core sample (sandsotne) and a synthetic brine formulated to replicate the composition of field-produced formation water, thereby reproducing realistic reservoir conditions. Results indicate that limestone exhibited a 6% increase in porosity and a 57% reduction in permeability due to calcium mineral dissolution and the formation of preferential flow pathways (wormholes) identified by CT imaging. In contrast, sandstone showed no significant changes in porosity, permeability, or mineralogical composition following CO_2_ exposure. Brine analyses revealed greater CO_2_ dissolution at lower temperatures, promoting carbonic acid formation and enhancing mineral dissolution processes in limestone. Furthermore, CO_2_–oil compatibility tests showed increased oil viscosity, suggesting destabilization of heavy hydrocarbon fractions and potential asphaltene precipitation under immiscible conditions. These results demonstrate that the response of the rock–fluid system to CO_2_ injection is strongly controlled by mineralogical composition and suggest that sandstones are a better candidate for long-term carbon capture and storage processes, while limestones may present a greater risk of petrophysical alteration and loss of permeability.

## Graphical abstract

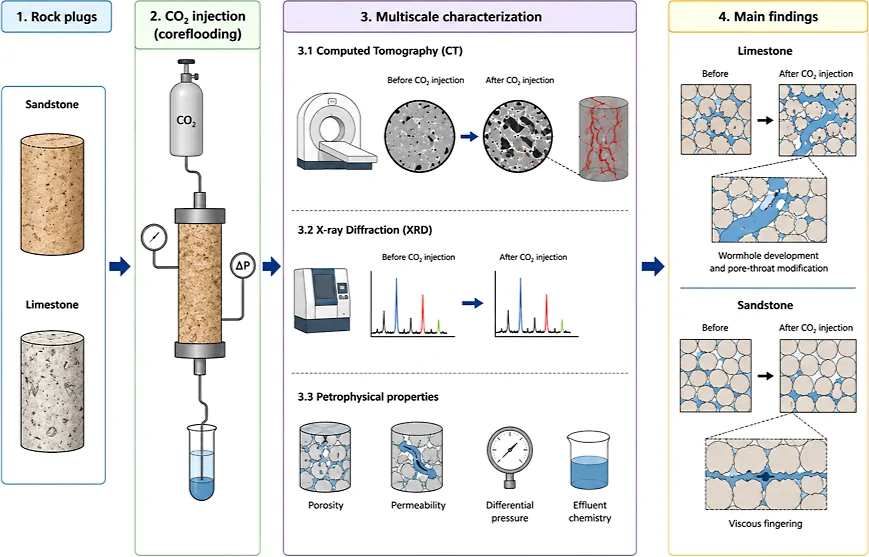

## 1 Introduction

High atmospheric levels of carbon dioxide (CO_2_) and their contribution to rising temperatures and global warming have been the subject of numerous studies, as demonstrated by the results presented in the IPCC assessment reports.^1–3^ In this context, carbon capture and storage technologies are key to mitigating greenhouse gas emissions and meeting carbon neutrality targets.^4^ However, the viability of these technologies does not depend solely on the storage capacity of the reservoir. Injectivity, petrophysical properties, and the geochemical stability of rock–brine/oil–CO_2_ systems are critical pillars in preserving the reservoir during carbon dioxide injection.^5,6^

During the injection process, CO_2_ interacts with the brine and rock matrix through coupled processes of flow, transport, and geochemical reactions or alterations. Depending on pressure and temperature conditions, carbon dioxide can be in a subcritical or supercritical state, defined by its critical point (31.1 °C and 7.38 MPa), which controls its density, solubility, and the degree of acidification of the formation water.^7–10^ Under pressurized subcritical conditions, CO_2_ has lower density and higher compressibility, which controls its solubility in formation water. In contrast, under supercritical conditions, carbon dioxide has a density close to that of a liquid fluid, increasing its dissolution and transport. The dissolution of CO_2_ in brine promotes the formation of carbonic acid, reduces the pH, and intensifies the dissolution and mineral precipitation reactions that can alter the permeability and porosity of rocks.^11,12^

In carbonate rocks, these processes become more important due to the reactivity of carbon dioxide-brine and minerals such as calcite and dolomite. Studies have shown that the interaction between formation water, CO_2_, and carbonates leads to mineralogical instability, dissolving the rock matrix and generating preferential flow channels known as wormholes, which alter the flow distribution and heterogeneity of the reservoir.^12–15^ At an optimal velocity, carbon dioxide-saturated brine creates thinner wormholes. In addition, the CO_2_ injection rate is directly proportional to the percentage of mineral dissolution and, therefore, to the rapid propagation of wormholes.^12^

In contrast, sandstone formations, which are usually dominated by the stability of quartz, respond better to CO_2_ injection. However, the presence of other minerals such as clays and cements can alter wettability and storage capacity due to the possible mobilization of fines or mineral precipitation.^16–19^ The interaction between carbon dioxide, water, and rock also reduces the fracture resistance, compressibility, and elastic modulus of the formation, which is critical and can compromise the long-term safety of CO_2_ storage.^20^

In CCS (Carbon Capture and Storage) and CCUS (Carbon Capture Use and Storage) scenarios involving depleted hydrocarbon reservoirs, variables such as miscible conditions or changes in the rheological properties of the fluid are introduced, including an increase in viscosity associated with the destabilization of heavy fractions and the aggregation or precipitation of asphaltenes.^21^

A comprehensive assessment of the effects associated with CO_2_ injection requires experimental techniques that allow changes in fluids and rock to be characterized. X-ray diffraction (XRD) is a tool that allows the identification of mineralogical variations induced by geochemical reactions, while computed tomography allows the observation of the evolution of pore space before and after displacement tests. These techniques are also a source of temporal evolution of the petrophysical properties of rocks.^22^ Conducting tests to determine the behavior of fluids constitutes an experimental evaluation of driving processes such as CO_2_ dissolution by acidification, brine concentration, and future alteration of the rock’s pore space.^23^

This study experimentally evaluates the impact of carbon dioxide injection on sandstone and limestone through integrated petrophysical and physicochemical analyses, including mineralogical characterization, computed tomography, fluid–fluid compatibility tests, and displacement experiments on rock plugs. The main contribution of this research lies in its focus on Colombian reservoir conditions, using a sandstone sample, a synthetic brine representative of Mugrosa Formation, and oil from the same formation, enabling a realistic assessment of CO_2_–rock–fluid interactions. In addition, a limestone sample was evaluated to investigate the potential effects of CO_2_ injection on analogous carbonate lithologies present in Colombia as Rosablanca, Tablazo and Paja formation.

## 2 Study Area

The Middle Magdalena Valley Basin is a structurally complex intramountainous system that has favored the accumulation of clastic sediments and the formation of significant oil reservoirs.^24^ The geology of this basin has undergone both extensional and compressional processes throughout its geological history. From the Late Triassic to the Late Cretaceous, the region was subjected to extensional stresses, while the Late Jurassic marked its transformation into a rift zone with continental and marine deposits.^25^

The stratigraphy of the Middle Magdalena Valley Basin (VMM) consists of a clastic succession of Mesozoic and Cenozoic age, within which units of regional importance are recognized, including the La Paz, Esmeraldas, and Mugrosa formations, widely documented as reservoir intervals in different sectors of the basin.^26–28^

The Mugrosa Formation is divided into two main zones: zone E, characterized by lutites of various colors interbedded with coarse to conglomeratic sandstones, deposited in meandering river systems; and zone A, composed of stacked sandstone packages deposited in river channel belts, with interbedded floodplain shales, where paleosols and pedological profiles are common. Zone A constitutes the main hydrocarbon deposit. The thickness of the Mugrosa Formation varies from 1330 m in the south to less than 600 m in the north of the VMM, due to changes in the subsidence of the basin. Its estimated age corresponds to the Early Oligocene.^26^

## 3 Methodology

Experimental laboratory tests were carried out on rock samples in order to evaluate the petrophysical and physicochemical changes induced by the interaction of carbon dioxide. The methodology included petrophysical measurements, displacement tests, mineralogical characterization, and physicochemical analyses of brines and oil under controlled pressure and temperature conditions.

### 3.1 Rock Samples

A sandstone sample and a limestone sample were selected for their representativeness within the study area. The outcrop sandstone sample, with a diameter of 3.76 and a length of 6.35 cm, was used as an analogue for the Mugrosa Formation, the main reservoir unit in the Middle Magdalena Valley Basin, in order to evaluate petrophysical variations associated with CO_2_ injection in clastic systems. The outcrop limestone sample, with a diameter of 3.79 cm and a length of 5.08 cm, was employed to analyze the response of calcite, which constitutes the rock matrix, to acidification processes induced by the interaction between CO_2_ and brine.

Porosity and permeability measurements were performed on both samples before and after the displacement tests in order to quantify the petrophysical variations resulting from the interaction between carbon dioxide and fluids (Figure 1). In addition, computed tomography analyses were performed to characterize the evolution of the pore space and the redistribution associated with mineral dissolution phenomena.

**Figure 1:**
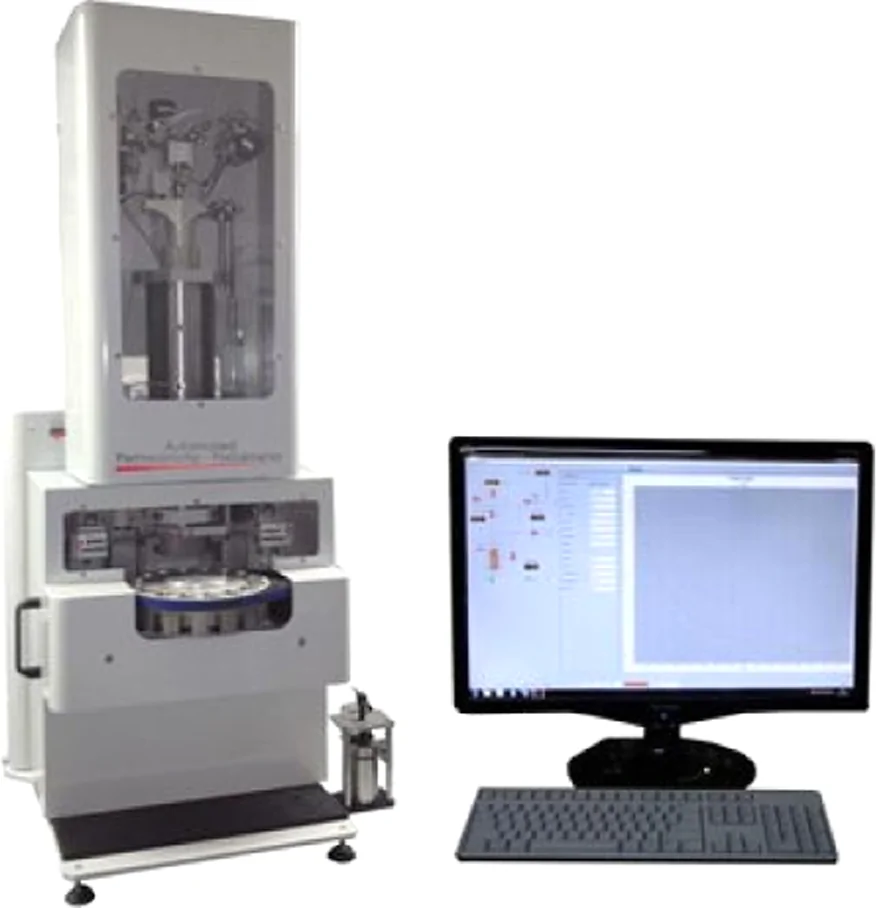
Image shows the KEYPHI apparatus, an automated instrument used to evaluate the properties of plug-scale core samples. Permeability determination is based on the unsteady-state pressure decay method. Pore volume is determined using the Boyle–Charles law technique.

### 3.2 Fluid Samples

Based on the geochemical and physicochemical characterization of the formation water in the Middle Magdalena Valley Basin,^29^ two representative synthetic brines were selected to evaluate fluid–fluid and rock–fluid interaction under CO_2_ injection conditions. These brines were named Brine 1 and Brine 2 in Table 1.

**Table 1:** Physicochemical Properties of Brines^a^

property	brine 1	brine 2
pH	6.15	6.17
TDS (g/L)	11.96	23.9
conductivity (mS/cm)	19.17	38.9
salinity (%)	11.9	24.7
resistivity (Ω·cm)	52	25.7

a Note: Brine 1 and 2 were developed synthetically.

Brine 1 was formulated to reproduce the average composition of formation water in Colombian sedimentary basins and was used during the displacement test or coreflooding of the limestone sample. In contrast, Brine 2 corresponded to a brine with a total dissolved solids concentration of 25,000 ppm, formulated based on the analysis of formation water from Field X, located in the Mugrosa Formation of the Middle Magdalena Valley,^29^ and was used in the coreflooding test of the sandstone sample.

During the CO_2_–brine and CO_2_–oil compatibility tests, the physicochemical variations induced by exposing the fluids to different pressure and temperature conditions were evaluated. These analyses made it possible to identify the changes associated with CO_2_ dissolution, acidification of the aqueous medium, and modification of the rheological properties of the oil.

The oil–CO_2_ compatibility test was carried out using a oil sample from Field X of the Mugrosa Formation, Middle Magdalena Valley Basin, which was also used in the coreflooding displacement test of the sandstone sample.

In order to evaluate the behavior of a rock–brine–oil–CO_2_ system under reservoir conditions, the sandstone sample was subjected to brine and oil from the Middle Magdalena Valley, whose properties are described in Tables 1 and 2, respectively. This experimental configuration allowed for an integrated analysis of the effects of CO_2_ on fluid–fluid and rock–fluid interactions, as well as its potential impact on the petrophysical response of the porous medium.

**Table 2:** Initial Properties of Oil^a^

property	value
BSW (%)	0
gravity API (°API)	24.9
dynamic viscosity (cP) a 30 °C	49.63
dynamic viscosity (cP) a 40 °C	33.55
dynamic viscosity (cP) a 50 °C	23.58
dynamic viscosity (cP) a 60 °C	9.8

a Note: Oil samples obtained from the Middle Magdalena Valley Basin.

### 3.3 Coreflooding Displacement—Experimental Configuration

Two coreflooding displacement tests were carried out under different experimental configurations. In the first test, the limestone sample was exposed to carbonated brine previously characterized as Brine 1 in Table 1. In the second test, the sandstone sample was subjected to interaction with brine (Brine 2) and oil from the Middle Magdalena Valley, according to the initial characterization in Table 2.

The configuration of the coreflooding displacement test performed on the limestone sample is shown in Figure 2 and was carried out in three sequential injection stages. In the first stage, the sample, previously saturated with Brine 1, was installed in the coreholder of the equipment and the absolute permeability was determined under ambient temperature conditions. Subsequently, brine was injected for 5–10 pore volumes (PV) until the differential pressure (DP) stabilized.

**Figure 2:**
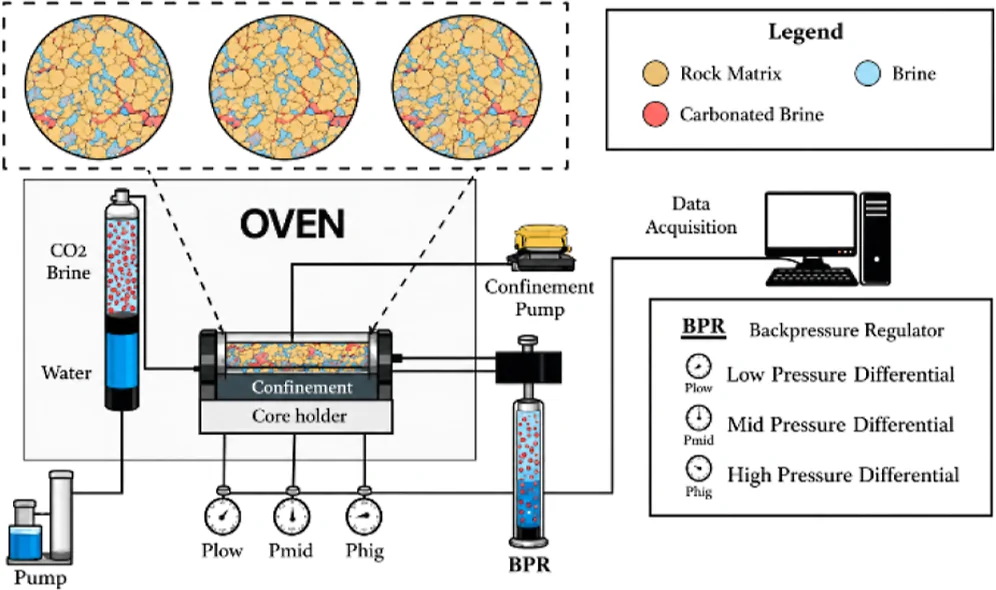
Coreflooding configuration in limestone. The image shows the configuration of the coreflooding displacement in the limestone sample, together with the propagation of the carbonate brine within the rock. Once the brine was placed into the cylinder, CO_2_ was injected to promote fluid mixing while the cylinder remained closed. Subsequently, the cylinder was integrated into the core flooding oven to increase its temperature and enhance the mixing process for 12 h.

In the second stage, the system was subjected to thermal expansion for 12 h using an oven integrated into the coreflooding system. The applied heat and pressure allowed the fluids and rock sample to behave under conditions representative of a real reservoir system. After stabilization, carbonated brine was injected at 120 °F, with a back pressure of 300 ψ and a confinement pressure of 1500 ψ. The injection flow rate in the test was 2 cm^3^/min. The injection was carried out until the differential pressure stabilized.

In the third stage, Brine 1 was injected under the same PVT conditions until the differential pressure reached final stabilization.

The coreflooding test performed on the sandstone sample shown in Figure 3 was carried out in four sequential stages. The sample was completely saturated with Brine 2 from Table 1 inside the coreholder. The absolute permeability was determined under ambient conditions by continuously injecting Brine 2 until DP stabilization was achieved.

**Figure 3:**
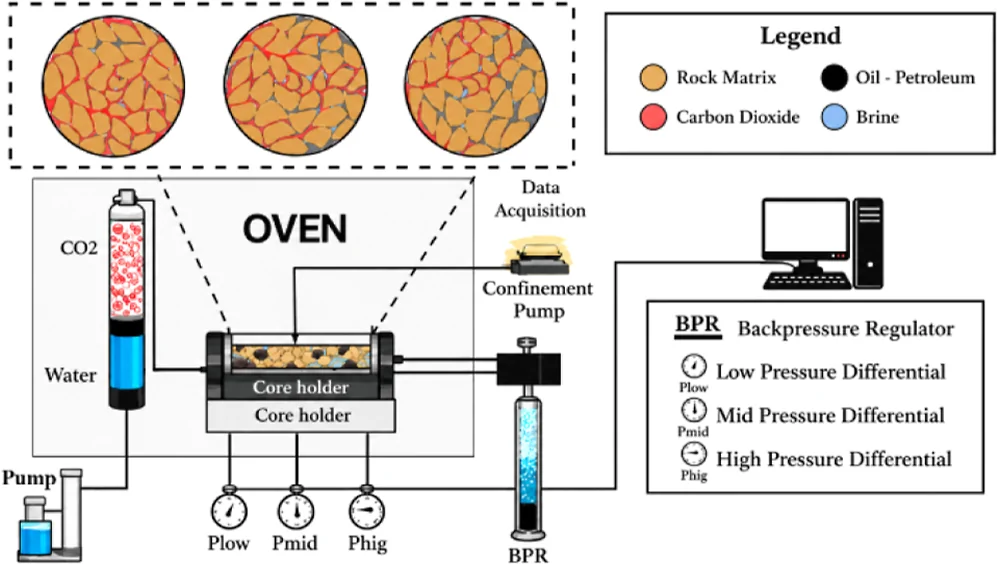
Coreflooding configuration (sandstone). The image shows the coreflooding displacement configuration in the sandstone sample, along with the propagation of CO_2_, oil, and brine within the rock.

The PVT conditions for the test were established (temperature of 150 °F, back pressure of 500 ψ, confinement pressure of 1500 ψ, and injection flow rate of 2 cm^3^/min for the aqueous phase and 2 cm^3^/min for the gas phase). The system was subjected to thermal expansion for 12 h. After stabilization, the absolute permeability was determined until a stable DP response was observed under the test conditions.

In the third stage, oil was injected until irreducible water saturation (Swirr) was reached. Subsequently, the plug underwent a wettability restoration process for a period of 10 days. Once restoration was complete, effective permeability to oil was measured under conditions of irreducible water saturation and the established *PVT* conditions.

In the final stage, CO_2_ gas was injected under test conditions. The effluent fluid from the test was collected continuously as the test progressed, allowing the effective permeability to CO_2_ to be determined under residual oil desaturation conditions.

### 3.4 Oil–Brine–CO_2_ Test

Compatibility tests were carried out to evaluate the physicochemical effects associated with the interaction of carbon dioxide with brine and oil representative of the reservoir. For each test, a 1000 mL high-pressure cylinder was used, into which 100 mL of representative fluid (brine or oil) was introduced.

The cylinders were pressurized while containing CO_2_ and brine using a high-pressure pump located outside the system. Subsequently, they were placed in a specialized oven to ensure proper thermal control, as shown in Figure 4 and in the process diagram in Figure 5. The tests were performed at 1000 and 3000 ψ and 130 °F and 170 °F, keeping the system static and in equilibrium for a period of 72 h. These conditions allow the behavior of the system to be evaluated under subcritical and supercritical regimes.

**Figure 4:**
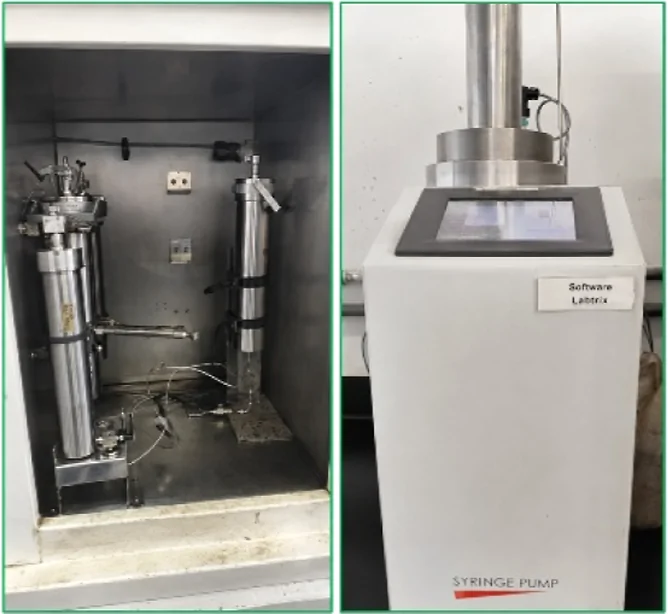
Assembly of the test in the oven and Vinci pump. The image on the left shows the compatibility test being carried out in the oven, together with four free-piston cylinders. The image on the right shows the Vinci pump, which provided the pressure in the system.

**Figure 5:**
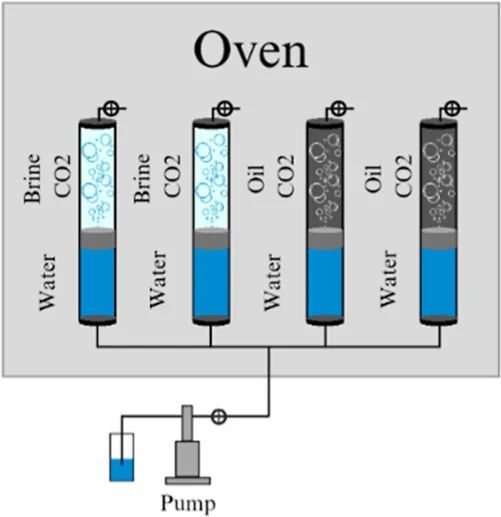
Oil–brine–CO_2_ compatibility test setup. The diagram shows the experimental setup for the compatibility test, using four distributed free-piston cylinders for the oil and brine.

Once the exposure time had elapsed, the system was depressurized in a controlled manner and the physicochemical properties were determined after the test. Table 7.

**Table 3:** Brine Composition^a^

ion concentration (mg/L)	brine 1	brine 2
Na^+^	3671.89	18932.57
K^+^	80.19	571.23
Mg^2+^	96.73	573.37
Ca^2+^	426.42	4469.48
Sr^2+^	-	153.25
Ba^2+^	-	233.6
Fe^2+^	-	1.46
Si	-	14.93
SiO_2_	-	34.12
Cl^–^	7440.68	1351.01
TDS	11715.91	24984.01

a Note: Brine 1 and 2 were developed synthetically.

**Table 4:** Required CO_2_ Volume

	required volume—*V*_2_ (cm^3^)	
temperature (°F)	1000 ψ	3000 ψ
130	68.06	75.30
170	57.20	64.73

**Table 5:**
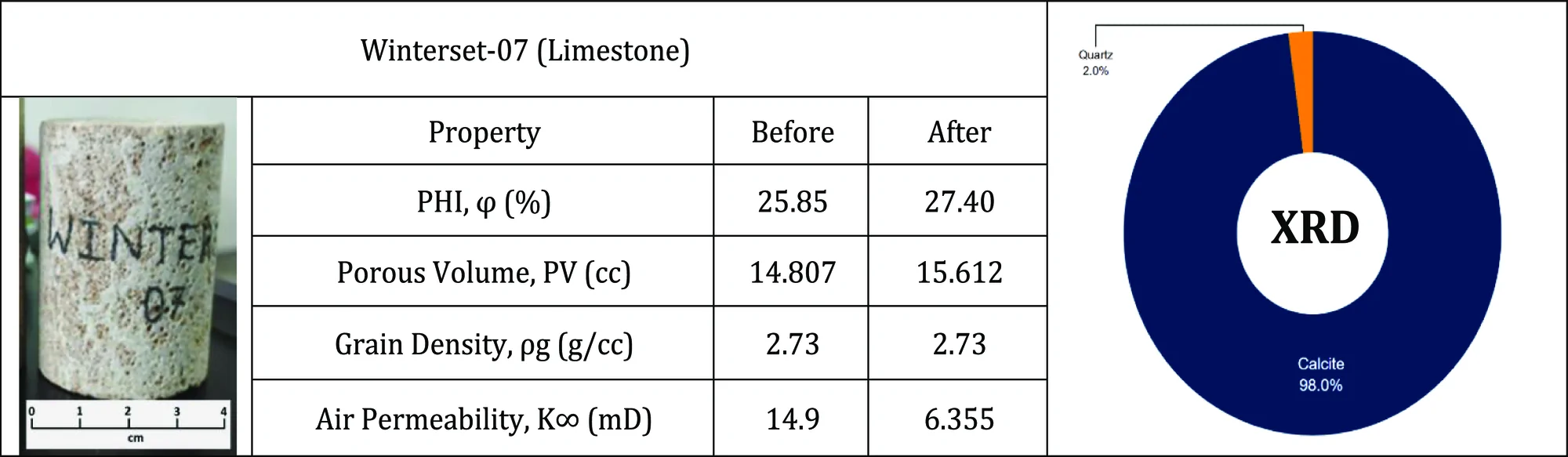
Petrophysical and Mineralogical Properties—Limestone

**Table 6:**
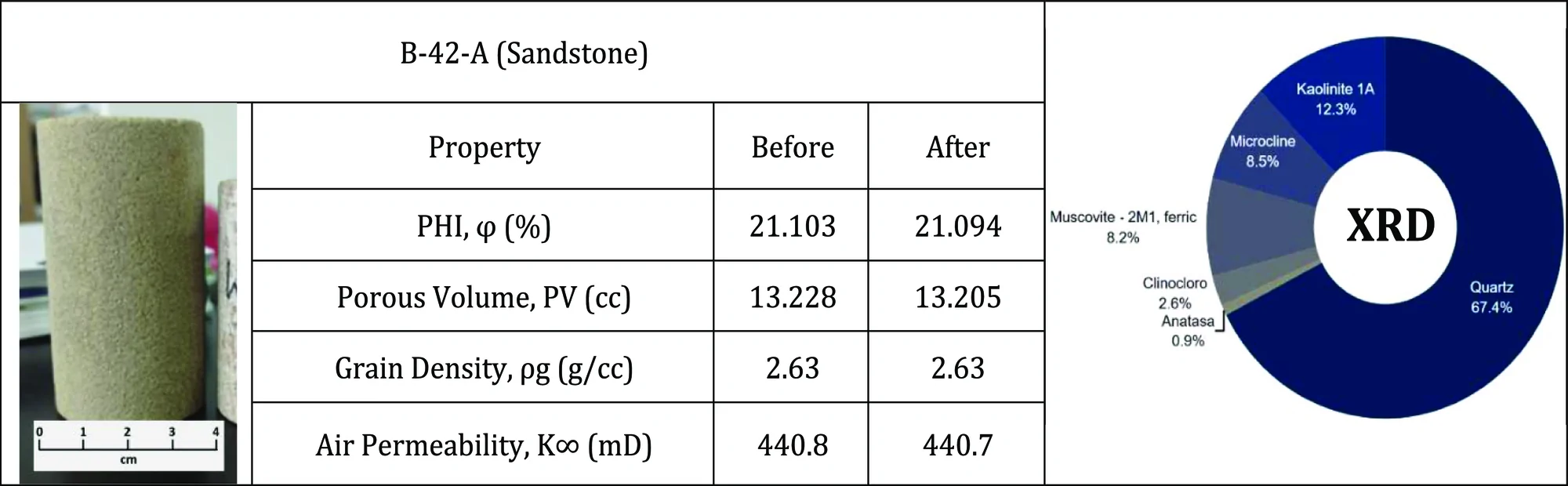
Petrophysical and Mineralogical Properties—Sandstone

The actual volume of CO_2_ present in the cylinders is calculated by simultaneously considering the effects of thermal expansion and gas compressibility under specific pressure and temperature conditions during testing. For this purpose, the general equation for real gases shown in eq 1 is used.

1 PV=ZnRT

where *P* corresponds to the system pressure, *V* to the gas volume, *Z* to the compressibility factor, *n* to the number of moles of CO_2_, *R* to the universal gas constant, and *T* to the temperature.

To determine the actual volume of CO_2_ under test conditions, two compressibility factors were calculated: *Z*_1_ corresponding to the pressure and temperature conditions of the test and *Z*_2_ corresponding to ambient conditions. Based on the conversion of the number of moles, the relationship expressed in eq 2 is established.

2 $$\frac{P_{1}V_{1}}{P_{2}V_{2}}=\frac{Z_{1}{nRT}_{1}}{Z_{2}{nRT}_{2}}$$

Rearranging the expression, the actual volume of CO_2_ is obtained using eq 3.

3 $$V_{2}=\frac{V_{1}Z_{2}T_{2}}{Z_{1}T_{1}}$$

where subscripts 1 and 2 refer to test conditions and ambient conditions, respectively. This procedure allowed the effective volume of carbon dioxide involved in the compatibility tests to be estimated. In particular, based on the relationship between pressure, volume, and temperature (*PVT*) described by the real gas equation, the volume *V*_1_, corresponding to the CO_2_ under test conditions, was determined from the results reported in.^32^ Additionally, the compressibility factor *Z* was evaluated using the known *PVT* conditions at both ambient and test states, with the aid of the interactive compressibility factor chart provided by the University of Colorado.^33^

## 4 Results and Discussions

### 4.1 Petrophysical and Mineralogical Analysis of Sandstone-Limestone

The initial and final properties of the samples are presented in Tables 5 and 6. In order to characterize the rock samples, petrophysical measurements of porosity and permeability were conducted. The porosity results for both limestone and sandstone samples have an uncertainty of ±0.33%. Likewise, the permeability measurements show uncertainties of ±0.011 mD for the initial limestone measurement, ±0.0019 mD for the final limestone measurement, and ±0.76 mD for both the initial and final sandstone measurements. In addition, X-ray diffraction (XRD) analyses were performed to determine the mineralogical composition of the samples.

After the coreflooding tests were performed, the sample exhibiting the greatest structural and petrophysical changes was the limestone, a result associated with the interaction of the CO_2_–brine system with a matrix mineralogically dominated by calcium carbonate/calcite. The concentrations of the ions present in each brine are shown in Table 3.

The interaction between carbon dioxide and brine generates the formation of carbonic acid,^11,12^ a process controlled by the solubility of CO_2_ under the pressure and temperature conditions of the test. Under the experimental conditions used, CO_2_ is present as pressurized subcritical carbon dioxide (Table 4). A fraction of the CO_2_ dissolved in the aqueous phase reacts with water to form carbonic acid, which induces a decrease in the pH of the system and promotes the dissolution of carbonate minerals, as seen in eqs 4–7.

4 $${CO}_{2\left(g\right)}={CO}_{2\left(aq\right)}$$

5 $${CO}_{2\left(aq\right)}+H_{2}O=H_{2}CO_{3\left(aq\right)}$$

6 $$H_{2}CO_{3\left(aq\right)}=H_{\left(aq\right)}^{+}+HCO_{3\left(aq\right)}^{-}$$

7 $$HCO_{3\left(aq\right)}^{-}=H_{\left(aq\right)}^{-}+CO_{3\left(aq\right)}^{2-}$$

The generation of hydrogen ions (H^+^) reduces the pH of the carbonated brine and promotes the dissolution of minerals such as calcite (CaCO_3_), the main component of the limestone sample. X-ray diffraction results indicate that this sample is composed of 98% calcite, which explains its high reactivity to mineral dissolution by carbonic acid.

After the coreflooding test, the limestone sample showed a 6% increase in porosity and a significant 57% reduction in permeability. The increase in porosity was due to the dissolution of the calcium carbonate matrix, which led to the development of secondary porosity and the generation of preferential flow channels. However, the simultaneous reduction in permeability indicates that dissolution processes do not necessarily improve effective pore connectivity.

The observed increase in porosity concurrent with a reduction in permeability following CO_2_ injection in limestone can be explained by coupled geochemical reactions and pore-structure evolution mechanisms. This behavior is interpreted as the result of a dissolution–precipitation process driven by the interaction between CO_2_-saturated brine and carbonate minerals. During injection, the dissolution of CO_2_ leads to the formation of carbonic acid, which promotes calcite dissolution and enhances local porosity, particularly in regions near the inlet face of the core, through pore enlargement and the generation of new void space.^34^ As the reactive fluid advances downstream, the progressive increase in Ca^2+^ and HCO_3_^–^ concentrations in the aqueous phase can lead to conditions of supersaturation with respect to calcite, favoring secondary mineral precipitation. Under this scenario, calcite precipitation preferentially occurs within pore throats and interconnecting channels, where even small mineral accumulations can significantly restrict fluid flow.

In despite the increase in total porosity, permeability evolution is not solely governed by pore volume, but rather by the connectivity, geometry, and distribution of the pore network. Pore-scale observations indicate that dissolution processes can induce structural heterogeneity (Figure 9), reduce effective pore throat sizes, and decrease the number of interconnected flow pathways, ultimately diminishing connectivity and increasing flow resistance.^35^ In addition, mechanisms such as fines migration and secondary mineral precipitation may partially or completely block critical flow conduits, further impairing permeability despite the creation of additional pore space.^35^ In carbonate systems, dissolution can also promote flow localization and wormhole development, where fluid preferentially travels through a limited number of dominant channels, effectively bypassing large portions of the pore network and reducing the overall hydraulic contribution of the matrix.^36^

**Figure 6:**
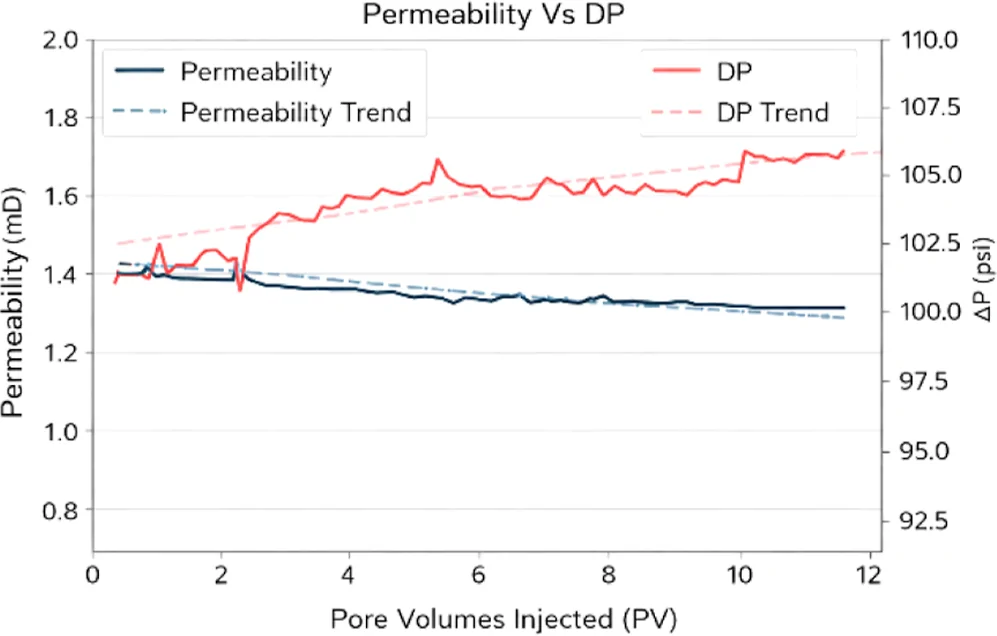
DP and permeability after CO_2_ injection. The graph shows the behavior and trend of the pressure differential and permeability with respect to the porous volumes injected with brine after the injection of carbonated brine into the limestone sample.

**Figure 7:**
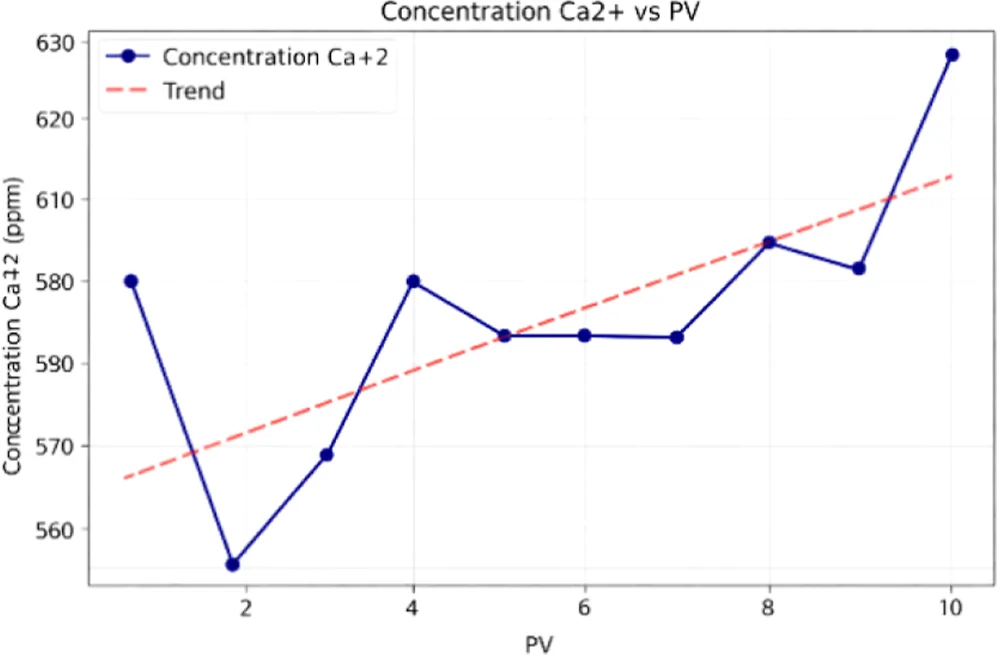
Calcium (Ca^2+^) concentration in effluents. The graph shows the measurement and trend of calcium concentration in carbonated brine effluents injected during the displacement of the limestone sample.

**Figure 8:**
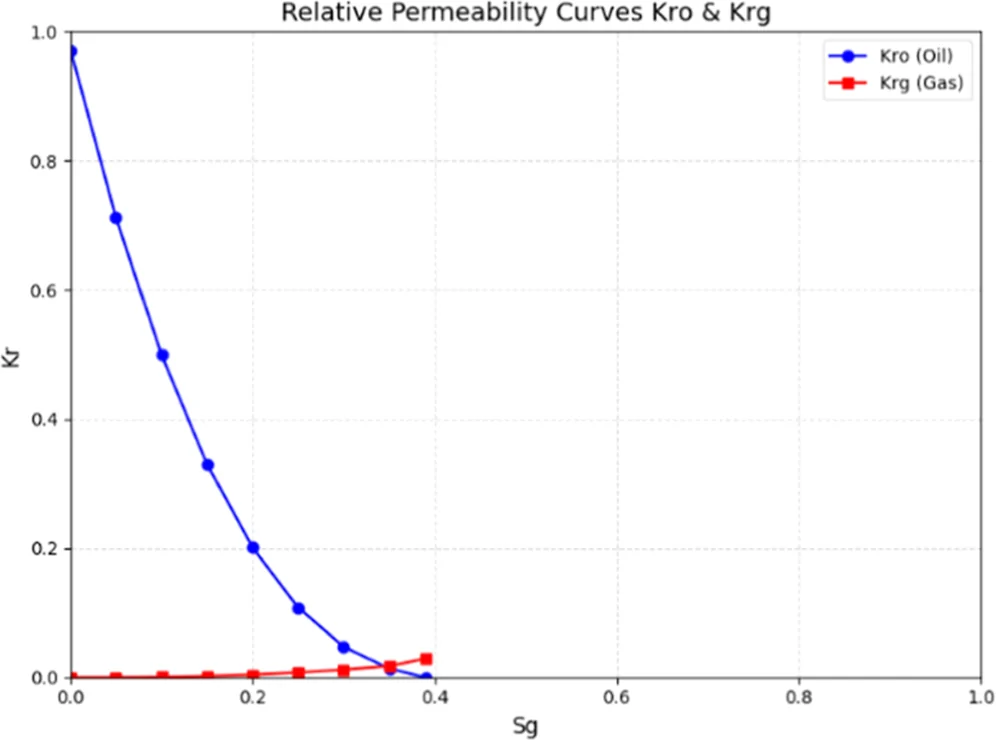
Relative permeability curves for crude oil and gas sandstone. The graph shows the behavior of the relative permeability curves between crude oil and CO_2_ at the moment of carbon dioxide injection.

**Figure 9:**
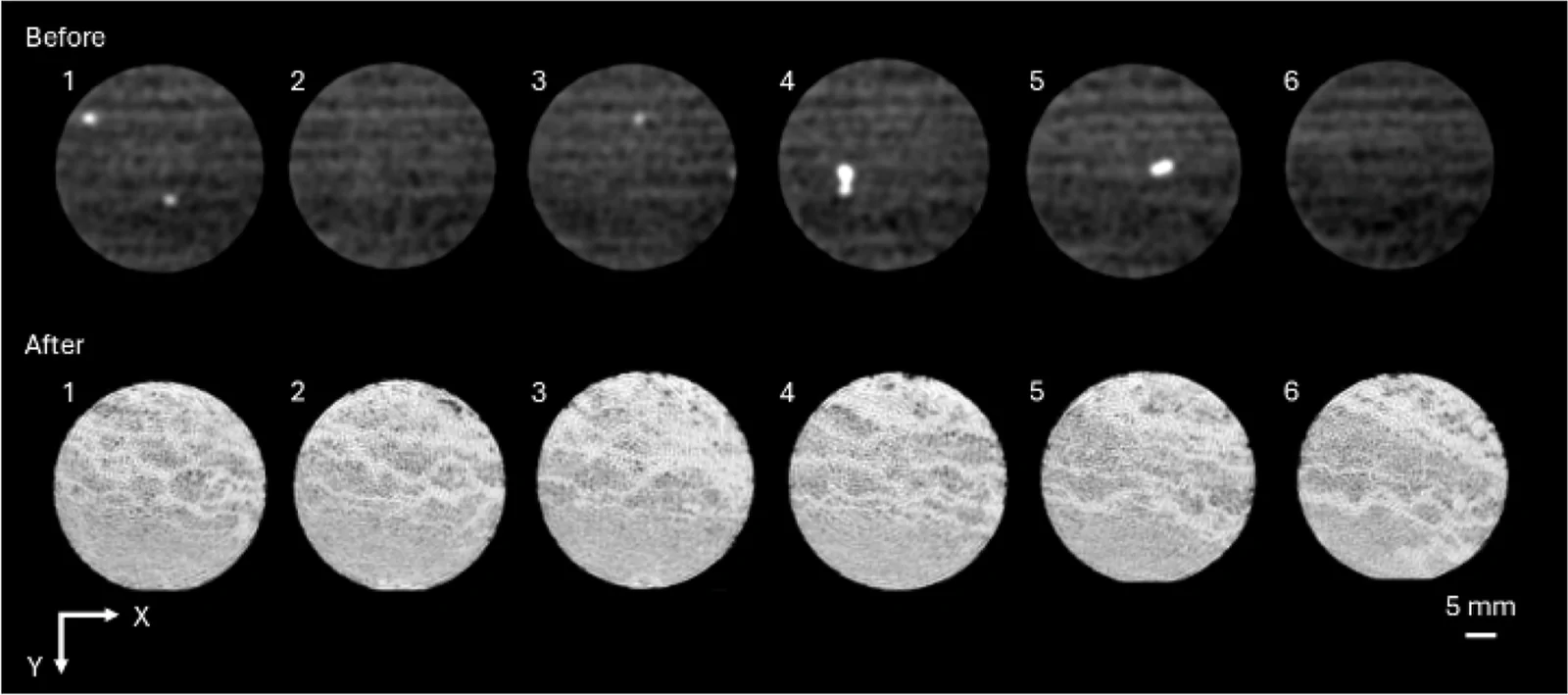
Berea limestone tomography (Winterset-07) before and after displacement. The tomography shows the different slices throughout the rock sample, providing a frontal view of the limestone.

In contrast, the sandstone sample has a mineralogical composition dominated by quartz, according to the crystalline phases detected in the XRD shown in Table 6, accompanied by accessory minerals and a small fraction of clays. Given the stability of quartz, CO_2_ did not induce significant mineral dissolution reactions in its matrix. The petrophysical properties measured after the coreflooding was completed show no appreciable variations in porosity or permeability. Furthermore, the homogeneous structure (Figure 10) of the sandstone matrix, as evidenced by the tomography images, together with its predominantly quartz-rich mineralogy, favored the development of preferential flow paths during CO_2_ injection. At the time of injection, the sandstone exhibited irreducible water saturation (Swirr) and a dominant oil saturation (So), conditions that promoted viscous fingering phenomena. This behavior is consistent with and supported by the relative permeability curves, which illustrate the instability of the displacement front and the preferential advancement of the injected CO_2_ phase through the porous medium.

**Figure 10:**
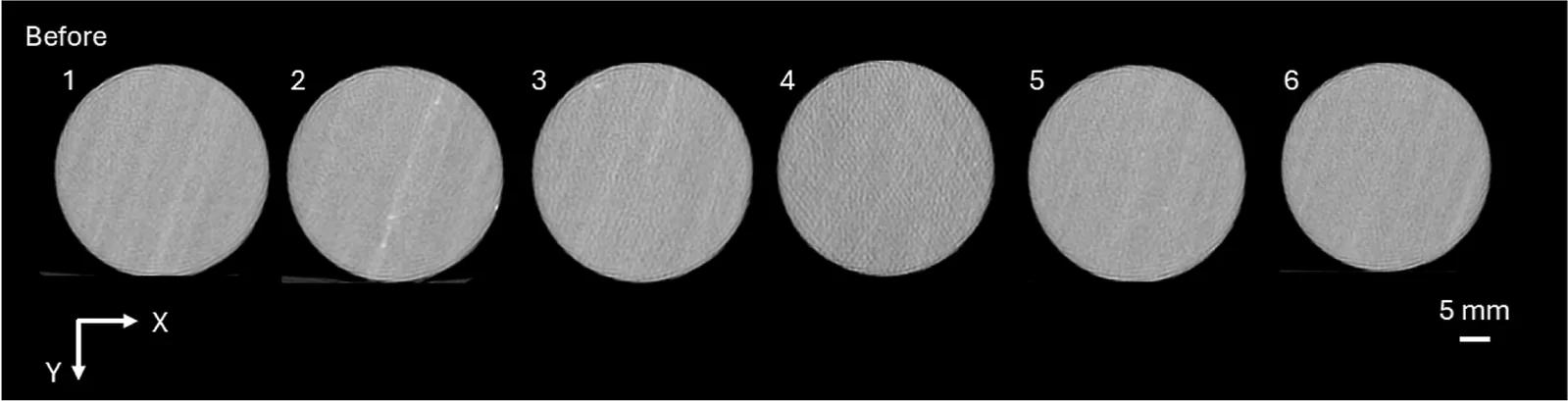
Berea sandstone tomography (B-42-A) before displacement. The tomography shows the different slices throughout the rock sample, revealing a homogeneous structure of the sandstone.

### 4.2 Coreflooding Displacement

The experimental procedure for carbon dioxide injection was achieved using coreflooding displacement equipment, which allows rock and fluid samples to be brought to pressure and temperature conditions that simulate the *PVT* conditions of a reservoir.

Once the injection of carbonated brine has been completed and the injection of brine without carbon dioxide has begun, the system reaches a stable flow regime, identified by the stabilization of the pressure differential. Under these conditions, variations in the permeability of the limestone sample are observed, determined from the behavior of the DP and Darcy’s law (Figure 6).

The progressive increase in the differential pressure (red line) indicates a reduction in the effective permeability of the core, as shown in Figure 6 with the blue line, consistent with the petrophysical results obtained after coreflooding. The dashed lines in Figure 6 indicate the trend of the DP and permeability data, respectively. Permeability shows a downward trend as porous volumes are injected. In the DP, the trend is upward, associated with the progressive loss of hydraulic connectivity of the porous medium.

During the experimental process, approximately 10 stable porous volumes of carbonated brine are injected through the limestone sample. Each porous volume was recovered as effluent and analyzed by determining dissolved calcium. The evolution of the calcium concentration is shown in Figure 7, which shows a trend consistent with mineral dissolution processes induced by the interaction between the carbonate brine and the calcium carbonate matrix of the core after the coreflooding test.

Figure 7 shows the evolution of dissolved calcium concentration in carbonated brine effluents as a function of the porous volumes (PV) injected during displacement. The dashed line represents the trend of the progressive increase in Ca^2+^ concentration in the effluent (mg/L). The fluctuations in the graph correspond to local heterogeneities in the porous medium and variations in the dissolution kinetics.

In contrast, the sandstone sample did not show significant variations in its petrophysical properties after the displacement test, maintaining a stable and continuous injection profile. In this case, carbon dioxide was not injected together with brine because the sample was saturated with crude oil in order for this displacement to simulate reservoir conditions. During displacement, the variables necessary for determining relative permeability curves were acquired using the Hirasaki method, as shown in Figure 8.

Analysis of the relative permeability curves indicates the presence of moderate interfacial tension between the phases involved, reflected in the slight concavity of the curves. This behavior is consistent with previous results reported on CO_2_–oil–brine systems, where interfacial tension controls the shape of the relative permeability curves and the efficiency of displacement.^30,31^ Under these conditions, interfacial interaction contributes to the instability of the CO_2_ displacement front by promoting the development of viscous fingering within the plug, a phenomenon mainly associated with the contrast in viscosities and densities between carbon dioxide and crude oil.

### 4.3 Brine–Oil–CO_2_ Compatibility

The viscosity of the crude oil was determined using a specialized rotational viscometer, which allowed the dynamic viscosity to be measured under different temperature conditions, ensuring precise control of the rheological parameters of the oil prior to compatibility measurements. The oil was subjected to a homogenization process using a controlled agitation system to ensure uniform distribution of its components. During this process, the equipment applies shear forces to the fluid according to the principle of momentum transfer.

Multiparameter equipment was used for the physicochemical characterization of the brine, determining properties such as pH, conductivity, resistivity, and total dissolved solids (TDS) before and after contact with carbon dioxide.

The results of the dynamic and rheological measurements of the crude oil after contact with CO_2_ are shown in Table 7.

**Table 7:** Crude Oil Viscosity Results before and after Contact with CO_2_—Compatibility Test^a^

fluid	30 °C	50 °C	60 °C	80 °C
before the test	49.63	23.58	9.8	-
oil 1000 ψ 130 °F	61.50	28.98	21.31	12.27
oil 3000 ψ 130 °F	69.76	29.59	22.17	12.93
oil 1000 ψ 170 °F	58.70	26.80	19.80	11.04
oil 3000 ψ 170 °F	69.90	32.84	23.61	13.33

a Note: Four compatibility tests were conducted under two pressure and temperature configurations.

During the initial stages of pressurization, carbon dioxide partially dissolves in the crude oil, inducing a volumetric swelling phenomenon associated with the incorporation of CO_2_ into the oleic phase. This process manifests as an increase in crude oil volume and an initial decrease in density, a behavior widely reported for CO_2_–crude oil systems under nonmiscible conditions. As system pressure increases, an extraction point is reached,^21^ defined as the pressure threshold at which CO_2_ begins to extract light hydrocarbons from the oleic phase into a CO_2_-rich phase.

The viscosity measurements obtained after the compatibility tests indicated that, once the extraction point is exceeded, the removal of light fractions reduces the swelling capacity and increases the effective density of the crude oil. Consequently, the enrichment in higher molecular weight components results in an increase in dynamic viscosity, a phenomenon reflected in the values measured after the compatibility test, as reported in Table 7.

Under pressurized subcritical conditions, CO_2_ partially dissolves in the crude oil, producing initial swelling and a moderate increase in viscosity. Under supercritical conditions, CO_2_ exhibits a greater dissolving capacity, leading to enhanced extraction of light hydrocarbons from the crude oil and causing a more pronounced destabilization of phase equilibrium, as observed in Table 8.

**Table 8:** Compatibility Test Results—Brine at 25,000 ppm

fluid	mS/cm	TDS	Sal	Ω/m	pH
before the test	38.9	23.9	24.7	25.7	6.17
1000 ψ	40.1	24.7	25.2	25.7	5.54
130 °F					
3000 ψ	43.2	24.75	25.2	23.14	5.38
130 °F					
1000 ψ	37.8	25	25.2	24.7	5.8
170 °F					
3000 ψ	39.45	25.02	25.2	25	4.82
170 °F					

In CO_2_–brine systems, carbon dioxide solubility is governed by the coupled interaction of physicochemical variables such as salinity, pressure, and temperature. An increase in salinity leads to a decrease in CO_2_ solubility, as Na^+^ and Cl^–^ ions reduce water activity and limit the availability of water molecules capable of dissolving the gas. In contrast, lower salinity levels increase the fraction of free water, promoting greater CO_2_ dissolution in the aqueous phase. This process involves the formation of carbonic acid and its subsequent dissociation, resulting in the release of ionic species and a reduction in pH following contact with carbon dioxide. Additionally, increasing system pressure enhances the density and effective solubility of CO_2_ in brine, intensifying acidification processes and the associated physicochemical changes.

### 4.4 Computed Tomography

Due to the mineralogical composition of the sandstone sample, no significant changes in its structure were observed. Therefore, based on the petrophysical and X-ray diffraction results, only data corresponding to the pre-CO_2_ injection stage and the displacement stage were obtained. The computed tomography (CT) images of this core plug are shown in Figure 11.

**Figure 11:**
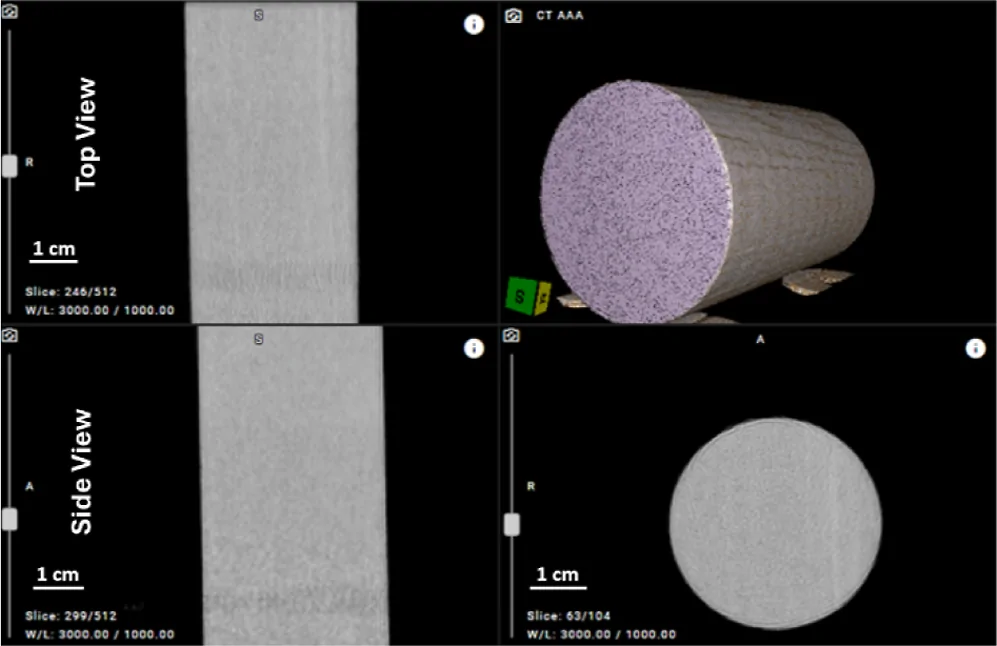
Berea sandstone tomography (B-42-A) before displacement. The tomography shows the different planes and the frontal view of the limestone sample.

The limestone sample underwent changes in its mineral structure after the displacement of carbonated brine, evidenced by the formation of wormholes, which generate preferential flow channels through which brine and carbon dioxide dissolve the calcium minerals in the rock. This phenomenon is shown in detail in Figures 12 and 13.

**Figure 12:**
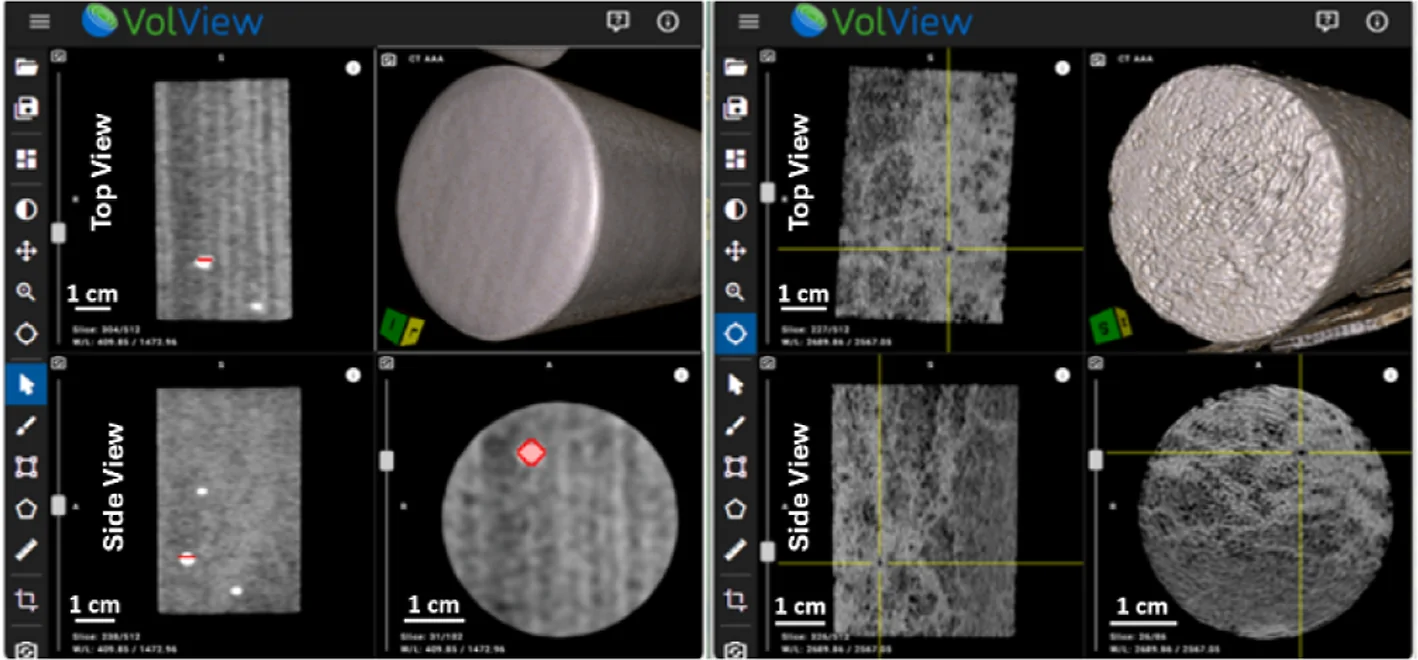
Comparison of limestone before and after displacement, respectively. The left tomography image shows the limestone sample prior to the injection of carbonated brine, while the right tomography image corresponds to the postdisplacement coreflooding stage.

**Figure 13:**
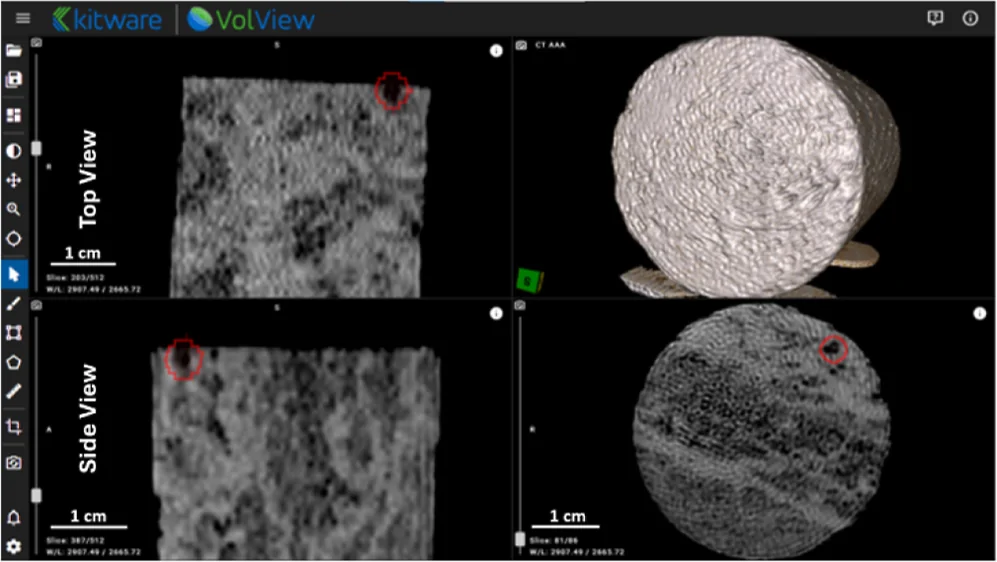
Wormhole detected after CO_2_ injection. The figure shows the initial creation of different wormholes on the plug face.

## 5 Conclusions

The petrophysical and geochemical response observed before, during, and after the tests indicates behavior dependent on the mineralogical composition of the rock and the properties and variables of the fluid-rock system. For sandstone, the petrophysical properties remain constant, indicating low reactivity of its mineral matrix under test conditions. Siliciclastic minerals, particularly quartz, provide greater stability against interaction with CO_2_. Additionally, the presence of internal laminations favors the development of preferential flow paths, limiting the volumetric saturation of the gas and promoting the appearance of viscous fingering. This behavior is associated with moderate interfacial tension and a contrast in viscosities between pressurized subcritical CO_2_ and crude oil, which explains the high residual oil saturation values and low effective gas saturation.

The limestone sample showed a moderate increase in porosity, close to 6%, after CO_2_ injection under displacement conditions with carbonate brine, suggesting slight mineral dissolution attributed to limited carbonic acid formation. This behavior is evidenced by the increase in calcium concentration measured in the porous volumes injected with carbonate brine. In contrast, permeability decreased by approximately 57% from its initial value, a result associated with the behavior observed in permeability and differential pressure after the injection of carbonate brine, as well as the precipitation of calcite in the interconnected channels of the rock and the injection flow rate used, equivalent to 2 cm^3^/min.

The variations observed in the compatibility test results for 25,000 ppm brine allow us to identify the solubility behavior of CO_2_ in this fluid. In particular, lower pH values are associated with greater CO_2_ dissolution. Based on this, it is determined that an increase in temperature reduces the solubility of CO_2_ in brine, which limits the reaction between the gas and the aqueous medium for the formation of carbonic acid. This phenomenon directly influences the dissolution of minerals sensitive to this acid, such as calcite. Conversely, high pressure values in the system favor an increase in the solubility of CO_2_.

According to the results obtained in the tests between oil and carbon dioxide, it was evident that, as the dissolved CO_2_ is released at the end of the test, a transfer of light components to the carbon dioxide-rich phase occurs. This migration of light fractions of crude oil is manifested in the increase in viscosity measured at the end of the compatibility test.

Overall, the results demonstrate that sandstones have greater potential for carbon capture and geological storage applications due to their petrophysical and mineralogical stability during CO_2_ injection, enabling large-scale and long-term storage projects while maintaining the capacity to securely store substantial amounts of CO_2_. In contrast, limestones show greater susceptibility to geochemical alteration processes that could compromise long-term permeability and injectivity.

## Data Availability

All data supporting the findings of this study are available within the article and its Supporting Information.
